# Deriving a Continuous Point of Departure for Skin Sensitization Risk Assessment Using a Bayesian Network Model

**DOI:** 10.3390/toxics12080536

**Published:** 2024-07-24

**Authors:** Fleur Tourneix, Leopold Carron, Lionel Jouffe, Sebastian Hoffmann, Nathalie Alépée

**Affiliations:** 1 L’Oréal, Research & Innovation, 1Eugène Schueller, 93600 Aulnay-sous-Bois, France; 2 Bayesia S.A.S., Parc Cérès, Bâtiment N 21, rue Ferdinand Buisson, 53810 Changé, France; 3 seh consulting + services, Stembergring 15, 33106 Paderborn, Germany

**Keywords:** skin sensitization, risk assessment, point of departure, Bayesian network, defined approach

## Abstract

Regulations of cosmetic ingredients and products have been the most advanced in embracing new approach methodologies (NAMs). Consequently, the cosmetic industry has assumed a forerunner role in the development and implementation of animal-free next-generation risk assessment (NGRA) that incorporates defined approaches (DAs) to assess the skin sensitization potency of ingredients. A Bayesian network DA predicting four potency categories (SkinSens-BN) was constructed against reference Local Lymph Node Assay data for a total of 297 substances, achieving a predictive performance similar to that of other DAs. With the aim of optimally informing risk assessment with a continuous point of departure (PoD), a weighted sum of the SkinSens-BN probabilities for four potency classes (non-, weak, moderate, and strong/extreme sensitizer) was calculated, using fixed weights based on associated LLNA EC3-values. The approach was promising, e.g., the derived PoDs for substances classified as non-sensitizers did not overlap with any others and 77% of PoDs were similar or more conservative than LLNA EC3. In addition, the predictions were assigned a level of confidence based on the probabilities to inform the evaluation of uncertainty in an NGRA context. In conclusion, the PoD derivation approach can substantially contribute to reliable skin sensitization NGRAs.

## 1. Introduction

Initiated by societal pressure and ethical concerns, political chemical safety programs around the globe are aiming at moving away from animal-based solutions and are calling for hazard and risk assessment approaches based on new approach methodologies (NAMs). Regulations of cosmetic ingredients and products have been the most advanced in abandoning animal experiments and embracing NAM solutions, especially in Europe [1,2]. Consequently, the cosmetic industry has assumed a forerunner role in the development and implementation of animal-free approaches to assess the primarily hazard of chemical ingredients.

Substantial efforts have been made to pave the way to advance NAMs for skin sensitization, which have led to substantial progress [3]. Based on the qualitative mechanistic understanding operationalized in the OECD skin sensitization adverse outcome pathway (AOP) [4], in vitro and in chemico test methods have been developed to address the first three key events (KEs) of the AOP. Based on thorough validation and independent assessment, several NAMs have been included in OECD Test Guidelines (TGs). TG 442C contains three in chemico reactivity test methods modeling the molecular initiating event (MIE) or KE1 “covalent binding of a chemical to skin protein” [5,6]: the Direct Peptide Reactivity Assay (DPRA), the Amino acid Derivative Reactivity Assay, and the kinetic DPRA (kDPRA), which were reviewed by Alépée, et al. [7]. TG 442D includes the KeratinoSens™ and the very similar LuSens, two in vitro test methods modeling KE2 “Keratinocyte activation” [8], while TG 442E features the four cell-based test methods U-SENS™, h-CLAT, IL8-Luc, and the GARD™skin that model KE3 “Dendritic cell activation” [9].

Although addressing the MIE could in theory be sufficient to cover a sequential chain of KEs, modeling errors inherent to the NAMs have led to the common understanding that a combination of NAMs covering at least two KEs of the skin sensitization AOP are required to provide high predictivity [10]. Consequently, a plethora of combinations of skin sensitization NAMs and also including other information sources, the so-called defined approaches (DAs), have been developed, including the sequential stacking tier strategy DAs developed somewhat later [11,12]. The majority of DAs have been reviewed by Kleinstreuer, et al. [13]. Recently, two DAs with a relatively simple decision tree approach to either predict skin sensitization hazard or United Nations (UN) Globally Harmonized System (GHS) classification categories have been adopted by the OECD [14]. The first three DAs included in this guideline use combinations of OECD-validated in chemico and in vitro test data, in some cases along with in silico information. The DAs currently described in this guideline are: (i) the “2 out of 3” (2o3) defined approach for hazard identification and (ii) two versions of the integrated testing strategy (ITSv1) for hazard and UN GHS potency categorization, both of which use the same test methods to address KE1 and KE3, but differ in the in silico predictions they incorporate (ITSv1: Derek Nexus; ITSv2: (OECD QSAR Toolbox 4.5)). The other Das have been developed for risk assessment purposes, i.e., they provide predictions of four categories or of a continuous value (see, for example, [13,15,16,17]). DAs can be applied as or transformed into a NAM-based point of departure (PoD) to be used in the next-generation risk assessment (NGRA) framework for skin sensitization, replacing the traditionally used animal-based or human-based PoDs [18,19].

Among them, a DA based on a Bayesian network, usually referred to as the Bayesian integrated testing strategy (ITS) for skin sensitization potency assessment, has been developed to derive a potency prediction of being a non-, weak, moderate, or extreme/strong sensitizer based on the Bayes factor [20]. Bayesian networks are probabilistic by definition, as they describe relationships between variables of the system of interest by conditional probabilities, which together form the joint probability distribution of the system. Bayesian networks can be graphically described by directed acyclic graphs (DAGs) comprising input variables, latent variables, and connections between dependent variables [21]. The advantages of Bayesian approaches have been acknowledged for risk assessment in general and for skin sensitization in particular, comprising the representation of the underlying mechanistic/dependent processes through DAGs, the ability to compensate for missing input data, and the inherent ability to address uncertainty [20,22,23,24,25]. In a risk assessment context, the selection of a category, either based on the maximum posterior probability or the Bayes factor, and subsequently of the lowest value in a category as the PoD, results in a limited number of potential PoDs. In addition, such a PoD comes with an unknown level of associated uncertainty, as the distance between the lowest category value and the true value can fall anywhere in the range that the category spans, as highlighted in a case study [18]. 

Building on the advantages of the Bayesian integrated testing strategy (ITS) for skin sensitization, a Bayesian network model (SkinSens-BN) was built to assess potency classification. This new DA offers enhanced flexibility by expanding the scope of the input data used and the covered chemistry (i.e., mainly cosmetic ingredients). While predicting four potency categories, which can be reduced to obtain predictions of UN GHS categories and skin sensitization hazard, the SkinSens-BN posterior probabilities were used to predict, in addition, a continuous PoD and to derive a categorical indicator of confidence associated with the predicted results.

## 2. Materials and Methods

### 2.1. Data Inputs

#### 2.1.1. NAMs

The 13 inputs included in the Bayesian network were selected to inform several biological events relevant for the skin sensitization mode of action. Building upon previous work (for example, summarized by [13,26]), bioavailability was addressed by three physico-chemical properties (molecular weight (MW) calculated from the structure, octanol–water partition coefficient (clogP) from Biobyte v.5.2, and volatility from EPISuite v. 4.1 categorized according to [27]), metabolism by Tissue Metabolism Simulator (TIMES-SS, v2.29.1.28 model v23.28), and the reactivity mode of action by the ToxTree module “Skin sensitization reactivity domains” (version 2.6.13) [28]. The three AOP key events were covered with the in vitro/chemico test methods DPRA, KeratinoSens™ [29] and the U-SENS™ [30,31], the latter two also informing cytotoxicity. Local Lymph Node Assay (LLNA) data extracted from the OECD database were used as a reference [32]. Further details are provided in Supplemental Table S1 “Inputs”.

In total, 297 chemicals were selected using pre-defined criteria, such as the availability of LLNA test results, which were used to determine the reference result for determining a substance’s potency. Data were retrieved from two sources: 219 substances, including 70 proprietary substances (identity not revealed) from Tourneix, Alépée, Detroyer, Eilstein, Martinozzi Teissier, Nardelli, Noçairi, Pauloin, Piroird and Del Bufalo [12], which included 184 cosmetic ingredients, inter alia, 72 dyes, 22 preservatives, and 40 fragrances; and 78 from the OECD database [32]. A total of 3861 input data were retrieved. NAM data were also collected from additional sources [12,33]. The data are summarized in Supplemental Table S1 “Data”. Training set input data were complete, except for 40 substances with inconclusive (12) or partly missing (28) DPRA data, one substance with partly missing KeratinoSens™ data, 14 substances with inconclusive volatility data, and 39 substances with inconclusive TIMES-SS predictions. Input data for the test set were complete, except for six substances not tested in the U-SENS™, four substances with inconclusive or missing DPRA data, four substances for which no or partly missing KeratinoSens^™^ data were available, and six substances with inconclusive TIMES-SS predictions. 

#### 2.1.2. In Vivo Reference Data

**Figure 1:**
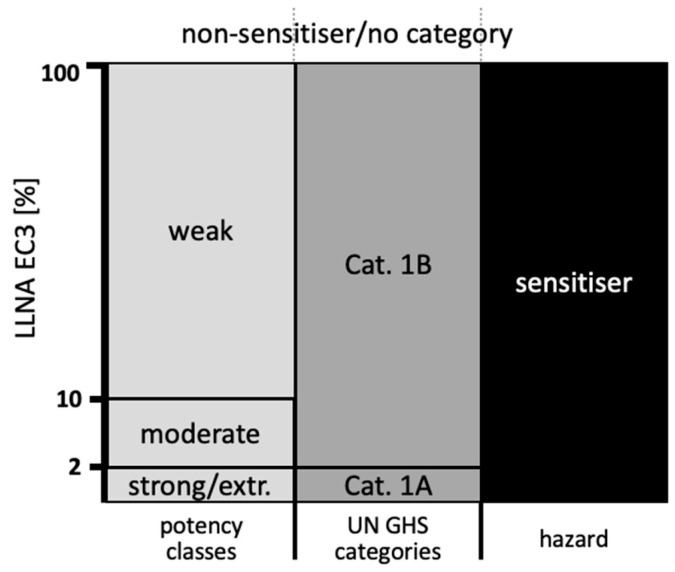
Relationship of skin sensitization categories for potency (4 classes), UN GHS categories, and hazard derived from LLNA EC3 values.

As the Bayesian network was to be constructed against reference data, Local Lymph Node Assay (LLNA) data were extracted from the OECD database [32]. Supplemental Table S1 “Data” includes the EC3 value for each substance, i.e., the interpolated dose that stimulates a three-fold increase in lymph node cell proliferative activity compared to the vehicle control. For non-sensitizers, the EC3 value was set at 100%, as conducted previously (for example, [18,34]). In addition, the skin sensitization categories of the UN GHS, i.e., Cat. 1A for EC3 ≤ 2% (strong/extreme sensitizers), Cat. 1B for EC3 between 2 and 100%, and no category (NS), and a 4-class categorization that divides the GHS Cat. 1B into weak (EC3 ≥ 10%) and moderate skin sensitizers (2% < EC3 < 10%), are presented (Figure 1) [35,36].

### 2.2. Bayesian Network

#### 2.2.1. Construction and Training

The Bayesian network was generated with BayesiaLab v10.2, a commercial software (available at https://www.bayesia.com (accessed on 17 July 2024)). The input parameters used are listed in Supplemental Table S1 “Inputs”, structured by the biological event they inform. Each quantitative input variable was discretized into two or three categories using by using a Minimum Description Length approach for finding the value of the thresholds and their number [37]. For the prediction of the four LLNA potency classes as described above, each quantitative input variable was discretized into two or three categories using data-driven thresholds. For example, the U-SENS™ EC150 thresholds were 3.86 and 51.79, resulting in three categories. All discretization thresholds for the quantitative input variables are provided in Supplemental Table S1 “Inputs”.

The SkinSens-BN network, based on data-driven and expert knowledge, is structurally similar to the one by Jaworska, Natsch, Ryan, Strickland, Ashikaga and Miyazawa [20], who described the rationale for the network structure, the direct dependencies, and the direction between nodes/inputs. The arrows connect the conditionally dependent variables. This representation allows to reduce the complexity of the network, i.e., the number of probabilities to estimate. Expert knowledge was used to create meaningful latent variables that allow to create probabilistic summaries of the associated manifest variables. The rationale of the selection of input variables that cover the three first key events of the AOP for skin sensitization and of the structure is provided in Supplemental Table S1 “Input”.

The 297 substances were divided into training and test sets based on the two major sources used for substance selection and data retrieval. The network was trained with the input data of 219 chemicals [11], which are provided in Supplemental Table S1 “Data”, to predict the posterior distribution of the probabilities of a chemical belonging to each potency class given the observations. The resulting network was tested with the 78 substances from the OECD database [32]. These were primarily selected to obtain the coverage of the LLNA potency spectrum and of the physico-chemical properties (MW, clogP, and volatility similar to that of the training set (Supplemental Figure S1)). However, the two sets differed in the proportion of UN GHS Cat. 1A substances (lower in the test set) and the clogP (higher in the test).

Four latent variables (bioavailability, metabolism, cysteine, and U-SENS™) were used to structure the network by connecting related inputs, e.g., the three physico-chemical inputs’ molecular weight, clogP and volatility informing bioavailability. The DAG representing the network was further structured manually. 

#### 2.2.2. Performance Analysis

The Skin-Sens-BN obtained with the training set was internally validated by 5-fold cross-validation, stratified to obtain identical distribution in the 4 LLNA potency classes. Cross-validation resulted in an average accuracy of 61%, which was considered to sufficiently demonstrate the robustness and generalizability of the predictive performance of the BN. 

The predictive capacity for four potency classes (non-sensitizer, weak, moderate, and strong/extreme), determined by the most likely predicted class, the three UN GHS (1A and 1B vs. No Category), and for skin sensitization hazard (NS vs. S) was assessed by comparison with the LLNA reference results using contingency tables. For a four-class prediction (NS/weak/moderate/strong–extreme), randomly assigning each item to a class would result in a 25% accuracy rate. In the current study, the achieved percentage surpasses this probability and even more the 50% chance of landing on either side for the binary S/NS prediction. As highlighted schematically in Figure 1, classes/categories are simply combined moving from potency to UN GHS to hazard. Pertinent predictive parameters, i.e., accuracy, and specificity and sensitivity for hazard classification were calculated. 

### 2.3. Confidence Categorization

In order to derive an indicator of confidence of a chemical belonging to a potency class, the predicted probabilities, called probability profile, were converted to Generalized Bayes Factors (GBFs) applying the same formula used by Jaworska, Natsch, Ryan, Strickland, Ashikaga and Miyazawa [20], i.e., calculating the ratio of the posterior odds and the prior odds in the training set per class. Subsequently, we transformed the GBFs to what was termed “weight of evidence” (W = 10 × log10 (GBF)), which is measured in deciban, with one deciban being “about the smallest change in W that is directly perceptible to human intuition” [38]. The confidence in the prediction was categorized based on the maximum W-values across the four potency classes. Based on Jeffreys’ decision rule [39], W-values between −5 and 5 were associated with “weak” confidence, W-values between −10 and −5 and between 5 and 10 with “moderate” confidence, and W-values smaller −10 or larger than 10 with “high” confidence.

In other words, the SkinSens-BN model provides for each substance an indication of the confidence in the predicted potency class result based on the data observation.

### 2.4. PoD Derivation

The final node of the Bayesian network returns the discrete probability p for a substance belonging to each of the four classes (non-sensitizer (NS), “weak”, “moderate” and “strong or extreme” sensitizer). Using fixed weights for each of the classes that were based on associated LLNA EC3-values, i.e., 100 for NS, 10 for weak, 2 for moderate, and 0.2 for strong or extreme) the point of departure (PoD) was defined as the following sum of weighted probabilities:

PoD_BN_ [%] = p(NS) × 100 + p(weak) × 10 + p(moderate) × 2 + p(strong or extreme) × 0.2 (1)

The weights relate to LLNA EC3-value, with 100 representing a non-sensitizing result in the LLNA, 10 representing to lowest EC3 considered to be weak, 2 representing the EC3 used to discriminate UN GHS categories 1A and 1B, and 0.2 considered as a representative value for strong and extreme sensitizer [36]. The PoD sum assumes its maximum when p(NS) = 100 and the other probabilities are 0. In this case, the PoD is 100, corresponding to a negative LLNA. The minimum is obtained for p(strong or extreme) = 100 (and the other probabilities being 0), resulting in a PoD of 0.2%. As this minimum is larger than the LLNA EC-value of 0.2%, the approach will, in comparison to the LLNA, systematically underpredict the PoD for highly potent sensitizers. This limitation is acknowledged, but considered acceptable, as substances with a low PoD are rarely used as cosmetic ingredients. On the other end of the spectrum, PoD_BN_ will practically always be <100%.

## 3. Results

### 3.1. SkinSens-BN and Its Predictive Performance

#### 3.1.1. The Network Structure

**Figure 2:**
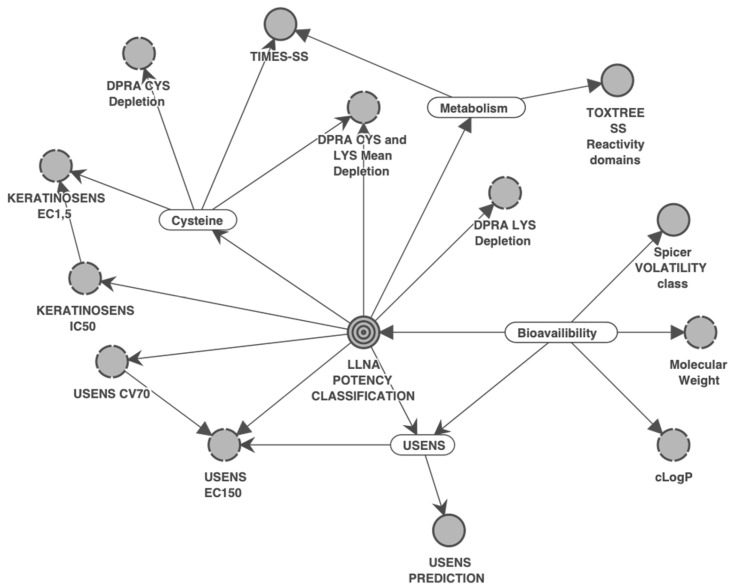
The directed acyclic graph (DAG) of the SkinSens-BN. The gray-shaded circles indicate the inputs (continuous border line: qualitative input; dotted border line: discretized quantitative input), rounded rectangles indicate latent variables, arrows connect dependent inputs, and the final node “LLNA potency prediction” is represented as a “target”.

The Bayesian network, referred to as SkinSens-BN, was constructed using 13 inputs informing various biologically relevant events, including mechanistic key events as operationalized in the skin sensitization AOP, and 219 defined substances, for which most inputs were available, and four latent variables. A graphical representation of the SkinSens-BN is shown in Figure 2. The inputs are displayed as circles. The four latent variables “Bioavailability”, “Metabolism”, “Cysteine”, and “U-SENS” are indicated as rounded rectangles, while the final node “LLNA potency prediction” is represented as a target. Arrows connect the dependent variables, with the arrow direction indicating the relationship.

#### 3.1.2. Predictivity: Training and Test Sets, Individual and Combined

The SkinSens-BN predictivity for the training set obtained by comparison to the LLNA reference data was calculated for discriminating four potency classes, the UN GHS categories, and a binary hazard classification. The results are summarized at the top of Table 1. The accuracy of prediction was 64% for the four potency categories, increasing to 68% for UN GHS categories and to 84% for hazard classes. For hazard, the specificity was 80% (55/69), the sensitivity 87% (130/150), and the balanced accuracy, i.e., the average of specificity and sensitivity, 84%. The number of substances over-predicted and under-predicted was very similar in each of the sub-tables, indicating the SkinSens-BN equally weighted mispredictions, i.e., not reducing mispredictions in one direction at the cost of the other directions.

In comparison to the training set, the test set predictions were lower for the potency classes and the UN GHS category, as indicated by reduced accuracies by 11% and 5%, respectively (Table 1, middle). The lower test set predictivity of the potency classes is primarily caused by the overprediction of weak sensitizers and mispredictions of moderate sensitizers. A potential reason explaining mispredictions is an imbalanced distribution of chemistry or other important factors between the training and test sets. For example, of the eight acrylates, three were in the training set and five in the test set, including four weak sensitizers that were overpredicted as strong/extreme. Note that three out of these four overpredicted weak sensitizers (GHS Cat. 1B) were also overpredicted as GHS Cat. 1A by both ITSv1 and ITSv2 [32]. Interestingly, the accuracy for hazard was higher (by 2%), as was the sensitivity (130/150 = 87% in the training set vs. 47/50 = 94% in the test set), while the specificity (55/69 = 80% in the training set vs. 20/28 = 71% in the test set) and balanced accuracy (83.2% in the training set vs. 82.7% in the test set) were lower. Table 1 also includes the predictivity when combining the training and test sets (at the bottom).

#### 3.1.3. Confidence Assessment

The confidence in the SkinSens-BN prediction was determined in relation to the maximum GBFs across the four potency classes, which were derived from the probability profile, i.e., the posterior distribution, and the prior. The maximum GBF was transformed into a W-value, which was interpreted using a simplification of the Jeffrey’s decision. The level of confidence was grouped into the three categories of high, moderate, and low. This level of confidence is intended to provide risk assessors with an indication of confidence when using SkinSens-BN results, informing the next risk assessment step. To summarize the results, these were grouped by potency category with the highest GBF. 

Of the 98 substances predicted as NS, the majority (71/98 = 72.4%) were associated with a “high” level of confidence. “Weak” substances were predominant (51/61 = 83.6%), with an assigned “moderate” level of confidence, as were substances with “moderate” potency (29/36 = 80.5%). The level of confidence of substances predicted as extreme/strong sensitizers was most evenly distributed, with a “high” level of confidence assigned to 48.0% (49/102) of the substances.

Table 2 exemplifies the approach of transforming the probability profile into a W-value, from which the level of confidence is derived by using three substances that were weak sensitizers in the LLNA. “Weak” was also the most likely predicted class in the SkinSens-BN probability profile. However, the individual probability profiles differ in shape, in particular the probability of the weak class (p(weak)). While p(weak) was very high for geraniol, it was just slightly higher than p(moderate) for hydroxycitronellal. This difference was also reflected in the W-values, leading to a high confidence for geraniol, a moderate confidence for N,N-dibutylaniline, and a low confidence for hydroxycitronellal.

### 3.2. Derivation of a Continuous PoD with SkinSens-BN and Its Comparison to EC3

**Figure 3:**
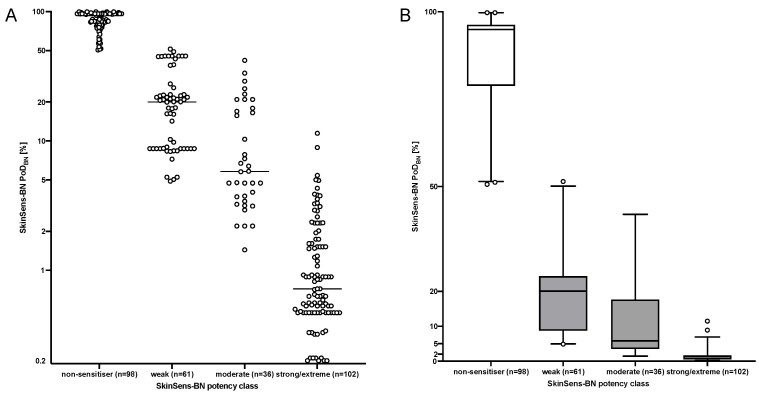
Comparison of PoD_BN_ (y-axis) and the predicted most likely potency class (x-axis), i.e., the class with the highest probability, with the number of substance (n) in the class: (**A**) as a dotplot (with median line) and (**B**) as a boxplot.

The discrete SkinSens-BN a posteriori probability distribution for the four potency classes, i.e., the probability profile, was used to construct an approach to derive a continuous PoD (PoD_BN_). For each of the 297 substances, a sum of these probabilities associated with fixed weights, which were based on LLNA EC3 values associated with each class, was calculated. The probability profiles for all substances and the PoD_BN_ are provided in Supplemental Table S1. The PoD_BN_ ranged from the absolute PoD minimum of 0.20%, which was obtained for four substances (lauryl gallate, tetrachlorosalicylanilide, dinitrochlorobenzene, and 4-nitrobenzyl bromide), to the maximum PoD_BN_ of 99.76% (Figure 3), with a median of 14.23%, a lower quartile of 1.52%, and an upper quartile of 78.84%.

To exemplify the approach, we selected three substances from the test set. Their identities, LLNA EC3 values, UN GHS category, probability profiles, predicted class, i.e., derived from the max. of the probability profile, and PoD estimates are summarized in Table 3.

For Lilial, all PoD (LLNA EC3, based on predicted class and PoD_BN_) were very similar. With an EC3 of 8.6%, the PoD_BN_ was almost identical (8.7%), while the PoD derived from the max. of the probability profile was 10%, i.e., the lower bound of the weak category. For 4-Methoxy-α-methyl benzenpropanal, the PoD_BN_ was similar to the EC3, both approx. a factor two higher compared to the approach of assigning it to the most likely potency class, i.e., a category PoD of 10%. For 3,4-Dihydro-coumarin, the class-based PoD and the EC3 were similar, while the PoD_BN_ was higher.

This comparison of the PoD_BN_ to the most likely potency class was conducted for all 297 substances. The results are summarized in Figure 3. Of the 297 substances, for 98, the most likely potency class was non-sensitizers. The PoD_BN_ in this class ranged from 50.62% to 99.76%, with a median of 94.94%, and was higher than all other PoD_BN_, except for one proprietary substance predicted in the weak potency class. The PoD_BN_ of the 102 substances in the strong/extreme potency class ranged from 0.20% to 11.47%, with a median of 0.7% and an upper quartile of 1.64%. The values clearly overlapped with the PoD_BN_ of the substances in the moderate potency class (33 substances), while the PoD_BN_ of 5 of those 33 overlapped with the weak potency class. Weak and moderate predicted potency classes, with medians 20.07% and 5.81%, respectively, showed wider distributions and overlapped considerably.

The large overlap indicated that potency grouping by the max. value in the probability profile may suggest a certainty in the result that is not reflected when considering the entire profile. 

Figure 3 also demonstrates that the PoD_BN_ will generally be higher than a PoD derived from the lowest threshold value of the predicted potency class. This is, for example, indicated by 22 substances in the strong/extreme potency class with a PoD_BN_ > 2%, including 1 substance with a PoD_BN_ of 11.5% and by only 1 substance in the moderate potency class with a PoD < 2%. In contrast, 21 of the 61 substances in the weak class had a PoD < 10% (min. of 4.89%). 

**Figure 4:**
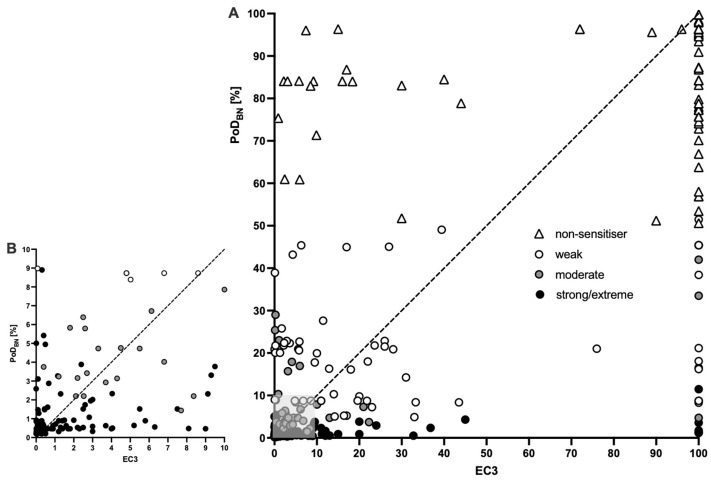
(**A**): PoD_BN_ (%) of the SkinSens-BN (y-axis) compared to the EC3 (%) from LLNA (x-axis) for the 297 chemicals. Shape and colors represent the potency category based on the max. value of the probability profile (diagonal line: line of identity). (**B**): Magnification of the gray-shaded area of (**A**).

Next, the continuous PoDs were compared with the corresponding LLNA reference EC3-values, assigning an EC3 of 100 to non-sensitizers. A dotplot of all PoD_BN_-EC3 pairs is shown in Figure 4. The data were clearly positively correlated, with highly statistically significant (*p*-values < 0.001) Pearson and Spearman correlation coefficients of 0.75 and 0.73, respectively, compared to the LLNA EC3. The data points below the line of identity indicate substances for which the PoD_BN_ is lower, and those above the line of identity indicate substances for which the PoD_BN_ is higher. For example, the cluster of data points (triangles) in the upper left corner of Figure 4A had a clearly higher PoB_BN_ and the substances with EC3-values between 2 and 10 were frequently overpredicted (black dots in Figure 4B low the line of identity).

**Figure 5:**
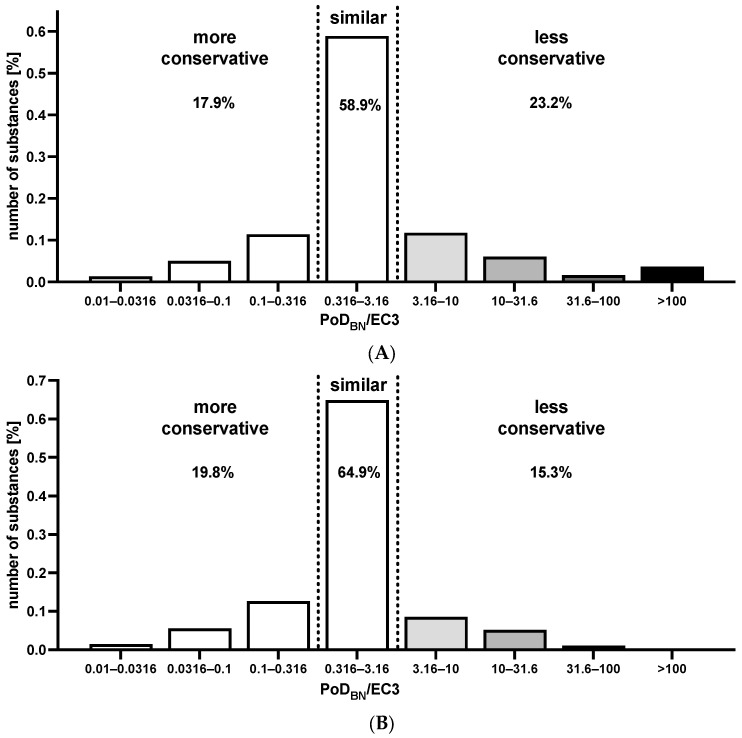
(**A**): Histogram of the ratio of the PoD_BN_ and the LLNA EC3 for the substances (*n* = 297). (**B**): Histogram of the ratio of the PoD_BN_ and the LLNA EC3 for substances with an EC3 ≥ 0.2 (*n* = 268). Substances with a ratio < 0.316 had a more conservative PoD_BN_ and substances with a ratio > 3.16 had a less conservative PoD_BN_ (gray shades).

To further quantify the similarity, the PoD_BN_/EC3 ratios were investigated. Values below 1 indicated substances with PoD_BN_ < EC3, i.e., more conservative-derived PoD, and values above 1 indicated substances with PoD_BN_ > EC3, i.e., less conservative-derived PoD. The ratios are represented as a histogram in Figure 5A. Ratios between 0.316 and 3.16, i.e., maximum 10^0.5^-fold difference in PoD_BN_ and EC3 in either direction, were considered as “similar”. This approach is based on the median LLNA EC3 standard deviation when using the same vehicle, i.e., 0.25, and the calculations presented by Hoffmann (2015). This group comprised 58.9% (175/297) of all substances. For 17.9% (53/297) of the substances, the PoD_BN_ was at least 3.16-times lower than EC3, i.e., more conservative, while the PoD_BN_ was less conservative for 23.2% (69/297). The occurrence of more severe less conservative ratios can partly be explained by the difference in the scaling of the two parameters at the lower end (minimum PoD_BN_: 0.2% vs. minimum EC3: 0.0003%). The least conservative PoD derived with the SkinSens-BN was derived for oxazolone, with a ratio of 6960 (20.88/0.003), which was also the most severely underpredicted substance in the linear regression-based PoD models by Natsch and Gerberick [15]. As a summary measure, the geometric mean fold error was calculated as 3.55 for all substances and as 3.97 for the test set only, indicating a slight decrease in performance.

To explore this effect of different scaling, a histogram without the substances with LLNA EC-values < 0.2, i.e., 29 most extreme skin sensitizers in the LLNA, is presented in Figure 5B. The absolute number of substances, for which the PoD_BN_ was more conservative, remained the same, i.e., 53, resulting in 19.8% due to the smaller total amount of substances. Except for a proprietary substance (OA39:EC3 = 0.1 and PoD_BN_ = 0.48), also the “similar” substances were not affected. However, as expected, the number of substances, for which the PoD_BN_ was less conservative, was substantially reduced by 28. It remains to be explored how to best address these scaling differences, e.g., by not deriving a PoD_BN_ for substances with a probability profile maximum value for the extreme/strong potency category that is associated with at the least moderate confidence.

## 4. Discussion

Skin sensitization is the human health effect for which a generally applicable, systematic, and exclusively NAM-based risk assessment approach is most advanced. An NGRA framework has been proposed that provides guidance for a tiered and transparent integration of relevant information, while allowing for flexibility [18,19]. This NGRA framework has been applied in various case studies to demonstrate its applicability and to initiate a constructive dialogue with stakeholders [22,40,41,42,43]. A centerpiece of the NGRA is the defined approaches (DAs) that integrate data from experimental NAMs that address at least two key events of the skin sensitization AOP [4]. Several DAs of different levels of complexity have been developed. These comprise two decision-tree-based DAs for hazard identification and classification according to the UN GHS, which were recently adopted by the OECD [14]. In addition, DAs using more sophisticated statistical approaches providing results that can, either directly or transformed, be used to derive a PoD for risk assessment have been proposed [20,44,45]. Among these, the Bayesian network by Jaworska, Natsch, Ryan, Strickland, Ashikaga and Miyazawa [20] is of particular interest due to its strengths, such as the provision of a probability profile across the potency classes, which can be used to quantify uncertainty associated with predictions, and the ability to cope with missing data. In contrast, the property that such network predictions are categorical limits the ability to derive a more precise PoD than the lower LLNA EC3-values associated with each class.

Building on the strengths, a Bayesian network similar to the one of Jaworska, Natsch, Ryan, Strickland, Ashikaga and Miyazawa [20] was developed, called SkinSens-BN. The main differences were the adaptation to some new input parameters and an increased total number of substances used that covered a broad spectrum of physico-chemical properties and LLNA EC3-values, including non-sensitizers. Overall, the predictive performance was considered in the range of the OECD-adopted DAs. The skin sensitization hazard was predicted as good, as reported for the ‘2of3′ DA [14,46]. Small differences in sensitivity and specificity were evened out, as indicated by the almost identical accuracies and balanced accuracies that ranged from 83% to 85%. In terms of UN GHS category predictions, the SkinSens-BN was compared to the “Integrated Testing Strategy (ITS)” DA, also included in the OECD TG 497. For both ITS versions, an accuracy of 71% was reported, with no misprediction over two categories and some inconclusive predictions. The respective accuracy of the SkinSens-BN was 67%. Six substances were mispredicted by two classes, five LLNA non-sensitizers as GHS Cat. 1A and one LLNA GHS Cat. 1A as a non-sensitizer (highlighted in Supplemental Table S1 “Data”). However, only one of those substances was part of the data used for calculating the ITS predictive performance in OECD TG 497. For these comparisons, it needs to be kept in mind that the number of substances was more than twice as high and that no inconclusive predictions were present for the SkinSens-BN. 

Regarding the prediction of four potency classes, which was 53% for the test set and 61% overall, a comparison with the Bayesian integrated testing strategy (ITS) for skin sensitization potency assessment would be most informative [20]. However, an independent evaluation is, to our knowledge, not available. An evaluation of the Bayesian integrated testing strategy (ITS) for skin sensitization potency assessment regarding three LLNA potency classes, which are similar to the GHS categories, obtained an accuracy of 68% with 115 substances, a predictivity very similar to the GHS predictivity of the SkinSens-BN [13]. Indications such as the seemingly lower predictivity for acrylates could be conducted as follow-up to further improve the SkinSens-BN, and may allow the further improvement in the predictive performance for four classes. However, it needs to keep in mind that the construction of the SkinSens-BN was primarily a means to an end. Nevertheless, the successful construction of a Bayesian network can contribute to building trust in the general approach and demonstrates the flexibility of the approach in terms of the inputs and chemistry to be covered.

Once it was confirmed that the SkinSens-BN performed promisingly, the primary goal of constructing an approach to derive a continuous PoD was addressed. A weighted sum combining fixed weights-associated LLNA EC3-values for each of the classes with the respective probabilities of their probability profile as weights was constructed. This approach, which is generally applicable, resulted in continuous PoDs in the theoretical range from 0.2 to 100%. The comparison of the SkinSens-BN PoD and the respective LLNA EC3-values showed that, for 77% of the 297 substances, a similar or more conservative PoD was derived. 

Options to further characterize and improve the SkinSens-BN include conducting a sensitivity analysis to characterize the impact that individual inputs have on the prediction, assessing the impact of missing inputs, and tuning it more toward human relevance, as conducted by Natsch [47]. The PoD_BN_ issue of underpredictions of strong and extreme sensitizers could be solved by the combination with an approach that can reliably identify extreme sensitizers in a first step, for example, by the use of NAMs targeting the identification of strong and extreme sensitizers, as the kDPRA [48], or by exploring targeted prediction models, as described for the ADRA by Alépée, Tourneix, Singh, Ade and Grégoire [7]. In addition, the adaptation of the PoD_BN_ algorithm and the adjustment of the PoD_BN_ based on analog information or a tiered decision process that specifically addresses extreme potency prediction, e.g., by not deriving a PoD_BN_ for substances with a probability profile maximum value for the extreme/strong potency category that is associated with at the least moderate confidence, could be explored. Although it needs to be considered that strong and extreme skin sensitizers are only very rarely, if at all, used as cosmetic ingredients, the improvement in an adapted strategy for these potency classes could be applicable to other industries, such as agrochemicals, botanicals, and medical and wearable devices, which are faced with the same concerns [49,50,51,52].

In addition, similarly to the approach of rating the confidence in predictions using the Bayes factor applied by Jaworska, Natsch, Ryan, Strickland, Ashikaga and Miyazawa [20], the probability profile was the basis to assign the prediction of confidence levels. The GBFs were transformed into W-values, which were categorized to provide the three confidence levels of “low”, “moderate”, and “high”. In essence, the approach resulted in a higher confidence the more probability was assigned to one of the four potency classes, or, in other words, the higher the distribution peak. On average, non-sensitizer potency predictions were associated with the highest confidence and moderate potency predictions with the lowest confidence.

The confidence rating is expected to substantially inform the next risk assessment steps in a weight of evidence approach. The overall risk assessment outcome is evaluated as a weight of evidence considering the calculated PoD [and, in the case of SkinSens-BN, the probability profile], the confidence in the use of NAM input data within the DAs (applicability domain), and the relative conservatism in the transformation of the DA outcome to a PoD (the most likely predicted class). For cases with insufficient confidence to reach a decision, it may, in combination with the detailed evaluation of individual inputs, point to the next steps in the process, which are needed to increase the confidence. Alternatively, the margin of exposure could be increased. This is intended to explore the usefulness of the SkinSens-BN-derived PoDs with associated confidence levels in several case studies, which may also identify potential avenues to improve the approach. 

In conclusion, the development of the SkinSens-BN model and its related PoD derivation approach, in the context of the NGRA, clearly indicates that quantitative risk assessments of skin sensitization can be achieved without a reliance on data from studies conducted on animals.

## Figures and Tables

**Table 1:** Contingency tables for training set predictions at the top and test set predictions in the middle and the combined sets at the bottom (gray shades: indicator of misprediction severity; bold: correct predictions; NS: non-sensitizer; mod.: moderate; extr.: extreme; S: sensitizer (weak/moderate/strong/extreme)).

Training Set				LLNA Reference Data Classes/Categories													
	**A: 4 potency classes**	NS	weak	mod.	strong/extr.	Σ		**B: UN GHS categories**	no Cat.	Cat. 1B	Cat. 1A	Σ		**C: hazard**	NS	S	Σ
**SkinSens-BN**	NS	**55**	12	7	1	75		NS	**55**	19	1	75		NS	**55**	20	75
	weak (EC3 ≥ 10%)	7	**20**	8	6	41		weak/mod.	11	**44**	12	67		S	14	**130**	144
	mod. (2% ≤ EC3 < 10%)	4	2	**14**	6	26		strong/extr.	3	23	**51**	77		Σ	69	150	219
	strong/extr. (EC3 < 2%)	3	5	18	**51**	77		Σ	69	86	64	219					
	Σ	69	39	47	64	219											
																	
	**accuracy**		64% (140/219)						68% (150/219)						84% (185/219)		
																	
**Test set**																	
	**A: 4 potency classes**	NS	weak	mod.	strong/extreme	Σ		**B: UN GHS categories**	no Cat.	Cat. 1B	Cat. 1A	Σ		**C: hazard**	NS	S	Σ
**SkinSens-BN**	NS	**20**	1	2	0	23		NS	**20**	3	0	23		NS	**20**	3	23
	weak (EC3 ≥ 10%)	6	**7**	6	1	20		weak/mod.	6	**20**	4	30		S	8	**47**	55
	mod. (2% ≤ EC3 < 10%)	0	2	**5**	3	10		strong/extr.	2	14	**9**	25		Σ	28	50	78
	strong/extr. (EC3 < 2%)	2	7	7	**9**	25		Σ	28	37	13	78					
	Σ	28	17	20	13	78											
																	
	**accuracy**		53% (41/78)						63% (49/78)						86% (67/78)		
																	
**Training and test set**																	
	**A: 4 potency classes**	NS	weak	mod.	strong/extreme	Σ		**B: UN GHS categories**	no Cat.	Cat. 1B	Cat. 1A	Σ		**C: hazard**	NS	S	Σ
**SkinSens-BN**	NS	**75**	13	9	1	98		NS	**75**	22	1	98		NS	**75**	23	98
	weak (EC3 ≥ 10%)	13	**27**	14	7	61		weak/mod.	17	**64**	16	97		S	22	**177**	199
	mod. (2% ≤ EC3 < 10%)	4	4	**19**	9	36		strong/extr.	5	37	**60**	102		Σ	97	200	297
	strong/extr. (EC3 < 2%)	5	12	25	**60**	102		Σ	97	123	77	297					
	Σ	97	56	67	77	297											
																	
	**accuracy**		61% (181/279)						67% (199/297)						85% (252/297)		

**Table 2:** SkinSens-BN probability profile of three example substances, with the corresponding W-values, predicted classes, and confidence levels.

Substance Name	Geraniol		N,N-dibutylaniline		Hydroxycitronellal	
LLNA EC3 (UN GHS cat.)	26% (1B)		19.6% (1B)		33% (1B)	
SkinSens-BN	Prob. Profile	W	Prob. Profile	W	Prob. Profile	W
p(NS)	0.1539	−4.02	0.0247	−1.26	0.0017	−2.43
p(weak)	0.7240	10.96	0.5687	7.97	0.3912	4.84
p(moderate)	0.1144	−3.38	0.2930	1.69	0.3810	3.41
p(strong/ext.)	0.0078	−1.72	0.1135	−5.08	0.2261	−1.45
predicted class	weak		weak		weak	
confidence	high		moderate		low	

**Table 3:** PoD_BN_ derivation for three example substances (bold: class with the highest probability).

Substance Name		Lilial	4-Methoxy-α-methyl benzenpropanal	3,4-Dihydro- coumarin
LLNA EC3		8.6%	23.6%	5.6%
UN GHS category		1B	1B	1B
SkinSens-BN probability profile	p(NS)	0.0244	0.1605	0.1614
	p(weak)	**0.5685**	**0.5185**	0.3946
	p(moderate)	0.2934	0.2736	**0.4284**
	p(strong/extreme)	0.1138	0.0474	0.0156
predicted class (PoD as lower bound of predicted class)		Weak (10%)	Weak (10%)	Moderate (2%)
confidence		moderate	moderate	low
PoD_BN_		8.73%	21.79%	20.94%

## Data Availability

The authors confirm that the data supporting the findings of this study are available within the article and its Supplementary Materials.

## Supplementary Materials

The following supporting information can be downloaded at: https://www.mdpi.com/article/10.3390/toxics12080536/s1, Figure S1: Comparison of EC3 values, GHS categories, and three physico-chemical properties of the training and the test sets using the Kruskal–Wallis test, with *p*-values; Table S1: Summary of all inputs used to construct the Bayesian network [5,27,28,29,30,31].
