# Supplementary material for: Deriving a Continuous Point of Departure for Skin Sensitization Risk Assessment Using a Bayesian Network Model
Source: Toxics. 2024 Jul 24;12(8):536. doi: 10.3390/toxics12080536 (PMC11360414; doi:10.3390/toxics12080536)

Supplemental Figure S1:

Comparison of EC3 values, GHS categories and three physico-chemical properties of the training and the test set using the Kruskal-Wallis test, with p-values.

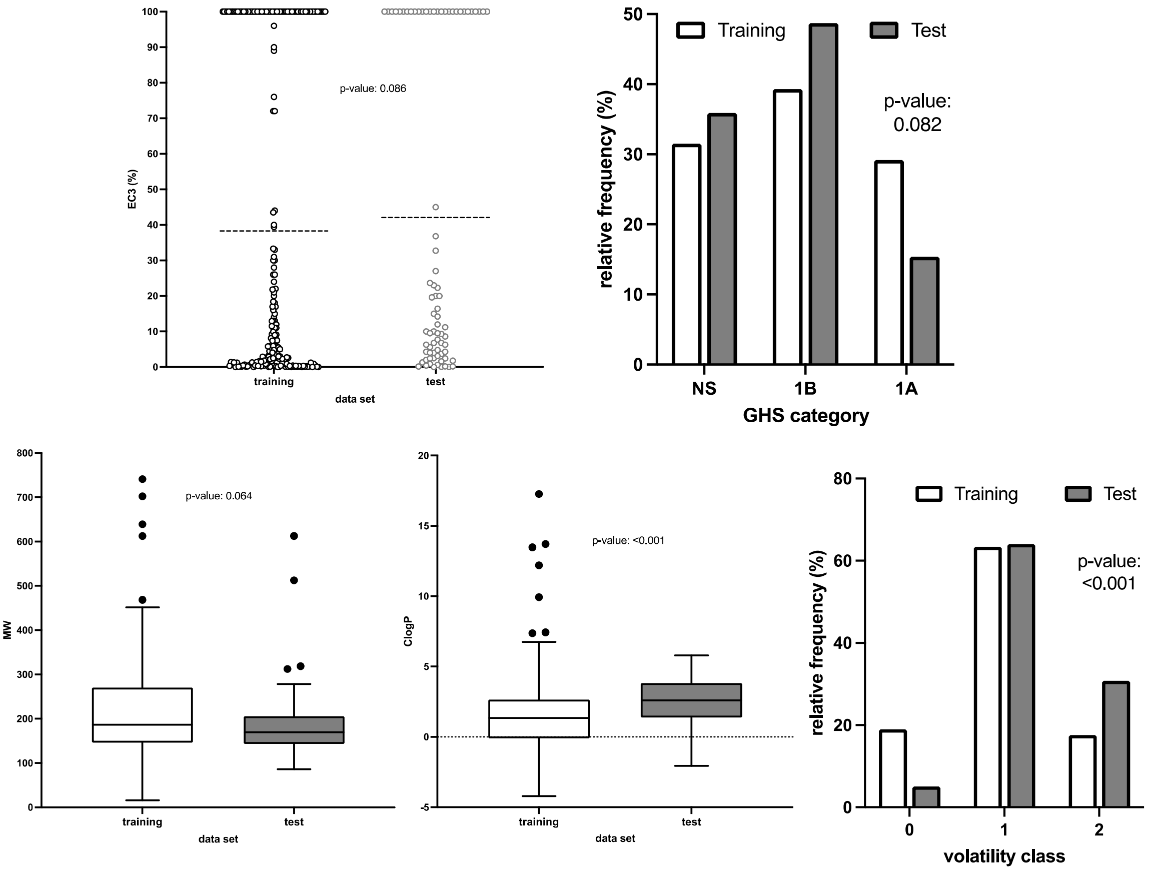

Supplement: Supplementary file 1 [file toxics-12-00536-s001.zip › Figure S1_FINAL.pdf]
